# Special Dental Pathology

**Published:** 1915-07

**Authors:** 


### BIBLIOGRAPHY

SPECIAL DENTAL PATHOLOGY. This is a new and the most important new book of the year. It is written by Dr. G. V. Black, Prof. of Dental Pathology in Northwestern University Dental School. Like Dr. Black's other works it is the result of his individual labors and not a compilation of the writings of other men. It is a most comprehensive treatise on the entire subject of dental diseases. It covers the whole realm of dental histology and pathology and has an excellent chapter on oral prophylaxis and hygiene. The work is the matured experience of a long life of research and practical experience as an operator and teacher of the subject, and as we should expect from a man of Dr. Black's caliber it represents the sum total of our knowledge on this subject from the clinical and research standpoints. It is orderly and systematically arranged as to the material and is thoroughly illustrated with many excellent pictures from microphotographs and drawings, for both of which Dr. Black has always had a high reputation for skillful productions. It is so extensive a volume, about 500 pages, that no conception of its real value can be given or even attempted in a brief review of this sort. We can only say that it appears to us to be the crowning glory of Dr. Black's great career as a teacher and scientific scholar in the dental profession. We are most fortunate that he has had the time and good health to complete this new and probably the last great work of this great scholar and devoted member of our profession. What this work has cost in labor and constant devotion, no one but his near friends will ever know. Books are so plentiful in this day that we do not stop to consider what they cost to those who write them. Several of our most brilliant men sacrificed their very lives to produce these necessary instruments for our professional progress. We honor our inventors or discoverers, but pass by our authors as though they were a mere matter of course, when as a matter of fact these are the men who have really made us a learned profession, and they should stand out as our greatest benefactors. No man should deserve more honor in this respect than the author of this newest and best of all our text-books.

Dr. Arthur Black has materially assisted his father in this great work, and the publishers are to be commended for producing the work in excellent form. It is published by the Medico-Dental Publishing Co. of Chicago.

